# Rapid and Sensitive Detection of Severe Acute Respiratory Syndrome Coronavirus 2 in Label-Free Manner Using Micromechanical Sensors

**DOI:** 10.3390/s21134439

**Published:** 2021-06-29

**Authors:** Dalal A. Aloraini, Aljawhara H. Almuqrin, Amal Alanazi, Qura Tul Ain, Abdullah N. Alodhayb

**Affiliations:** 1 Department of Physics, College of Science, Princess Nourah bint Abdulrahman University, Riyadh 11671, Saudi Arabia; daalorainy@pnu.edu.sa (D.A.A.); ahalmoqren@pnu.edu.sa (A.H.A.); 2 King Abdullah Institute for Nanotechnology, King Saud University, Riyadh 11451, Saudi Arabia; a.alanazi723@gmail.com (A.A.); qtulain1@outlook.com (Q.T.A.); 3 Research Chair for Tribology, Surface, and Interface Sciences, Department of Physics and Astronomy, College of Science, King Saud University, Riyadh 11451, Saudi Arabia; 4 Department of Physics and Astronomy, College of Science, King Saud University, Riyadh 11451, Saudi Arabia

**Keywords:** microcantilever sensor, DNA hybridization, SARS-CoV-2 detection, dynamic mode

## Abstract

Coronavirus (COVID-19), caused by the severe acute respiratory syndrome coronavirus 2 (SARS-CoV-2), has been identified as a deadly pandemic. The genomic analysis of SARS-CoV-2 is performed using a reverse transcription-polymerase chain reaction (RT-PCR) technique for identifying viral ribonucleic acid (RNA) in infected patients. However, the RT-PCR diagnostic technique is manually laborious and expensive; therefore, it is not readily accessible in every laboratory. Methodological simplification is crucial to combat the ongoing pandemic by introducing quick, efficient, and affordable diagnostic methods. Here, we report how microcantilever sensors offer promising opportunities for rapid COVID-19 detection. Our first attempt was to capture the single-stranded complementary DNA of SARS-CoV-2 through DNA hybridization. Therefore, the microcantilever surface was immobilized with an oligonucleotide probe and detected using complementary target DNA hybridization by a shift in microcantilever resonance frequency. Our results show that microcantilever sensors can discriminate between complementary and noncomplementary target DNA on a micro to nanoscale. Additionally, the microcantilever sensors’ aptitude toward partial complementary DNA determines their potential to identify new variants of coronavirus. Therefore, microcantilever sensing could be a vital tool in the effort to extinguish the spreading COVID-19 pandemic.

## 1. Introduction

The severe acute respiratory syndrome coronavirus 2 (SARS-CoV-2), which was identified in late 2019, has led to the coronavirus (COVID-19) pandemic [1,2]. The human-to-human transmissibility of SARS-CoV-2 substantially contributes toward worsening the ongoing pandemic [3]. Despite a global health emergency declared by the World Health Organization, approximately 2.9 million deaths because of the SARS-CoV-2 infection have been reported so far [4,5]. Insufficient testing is a major reason for the spread of SARS-CoV-2. Currently, real-time fluorescence reverse transcription-quantitative polymerase chain reaction (RT-PCR) testing is a reliable diagnostic method for detecting COVID-19 [6]. Because of its high sensitivity, large dynamic range, and specificity, RT-PCR testing is the mainstay of COVID-19 diagnosis. RT-PCR testing is based on detecting the specific viral ribonucleic acid (RNA) sequence responsible for COVID-19. The genome of SARS-CoV-2 is identified as positive-sense single-strand nonsegmented RNA (++ssRNA) having 27–32 kilobases [7]. Routinely, the purification of extracted RNA, reverse transcription to complementary DNA (cDNA), and analysis of amplified cDNA precede RT-PCR testing. To perform RT-PCR testing, specialized laboratories, practiced technicians, and several hours are needed, which is expensive and manually laborious [8,9]. Therefore, methodological simplification is crucial to control infection by increasing availability and efficiency. Several efforts have been made to circumvent RNA extraction in COVID-19 RT-PCR testing [9,10,11,12]. However, the presented methods still require DNA amplification, which is a major bottleneck in this procedure. A quick, efficient, and affordable diagnostic method is needed for early COVID-19 detection.

The fast response and ultra-sensitivity of microcantilever sensors ensure their remarkable application in bio-sensing [13,14]. Additionally, microcantilever sensors have been extensively used for monitoring biochemical interactions and detecting diverse types of viruses such as the human immunodeficiency virus [15], hepatitis C virus [16], hepatitis B virus [17,18], and feline coronavirus [19]. To achieve specificity and sensitivity, the microcantilever’s surface is immobilized with a specific probe or antibody that is responsive to the target molecule. The target molecules on the microcantilever surface are recognized by adsorption, which can induce a change in deflection or a shift in the resonance frequency of the microcantilever. Measuring the resonant frequency shift and deflection corresponds to the dynamic and static operation modes, respectively [13,20]. In the static operation mode, the change in microcantilever deflection is attributed to the surface stress induced by the molecular reaction on the microcantilever surface. Detecting biomolecular interaction in the static mode requires functionalizing only one surface of the microcantilever, which can be complicated, especially in an array of microcantilevers. Nonetheless, in the dynamic mode, functionalizing only one surface of the microcantilever is not an obligatory condition, as the resonance frequency shifts because of the total mass of the microcantilever [20,21]. In this mode, the microcantilever has a high sensitivity to the loading mass and serves as a microbalance. Therefore, detecting biomolecular interaction and viruses in the dynamic mode of operation could be a convenient approach. In this study, we demonstrate the label-free rapid detection of SAR-CoV-2 using the microcantilever sensor. The DNA hybridization technique is used to detect SARS-CoV-2 genomic sequences on the microcantilever sensor’s surface. To achieve this, a thiolated oligonucleotide probe was immobilized onto the gold-coated microcantilever surface and detected complementary target DNA hybridization by a resonance frequency shift in the dynamic mode of operation. Furthermore, we demonstrate the microcantilever sensor’s response to the complementary target DNA in the micro- to nanoscale. The immobilized microcantilever sensors, which are based on mechano-detection, can fully discriminate between the complementary and noncomplementary targets of SARS-CoV-2.

## 2. Materials and Methods

### 2.1. Microcantilever Chips

To conduct this study, Octo 500S/Au microcantilever chips were obtained from Micromotive (Germany). Each chip has an array of eight microcantilevers (500 ± 5 μm long, 90 ± 2 μm wide, and 1 ± 0.3 μm thick) with a Young’s modulus of approximately 140 GPa coated with Ti/Au thin film of 20 nm. Before probe (single-stranded DNA) immobilization, the chips were rinsed with piranha solution (3:1 sulfuric acid to hydrogen peroxide) to remove any organic contaminants.

### 2.2. Probe Immobilization

**Figure 1:**
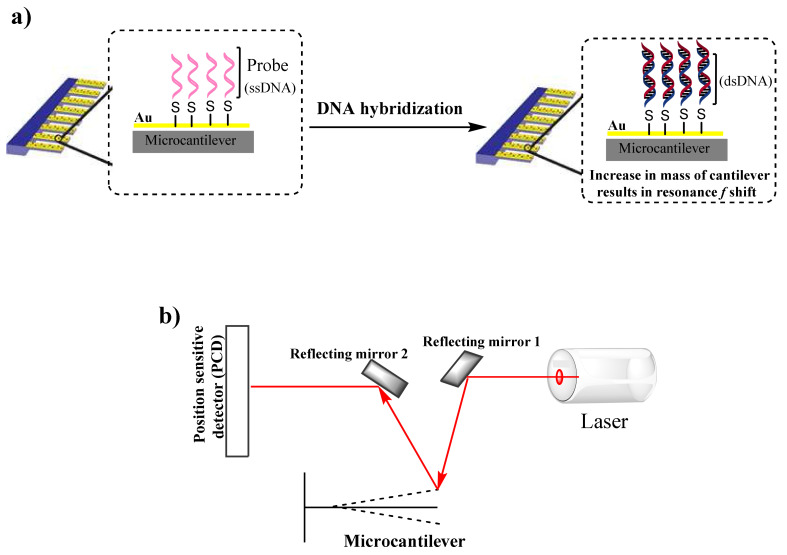
(**a**) DNA hybridization to immobilized microcantilever, which induces an increase in the microcantilever mass, resulting in a resonance frequency shift in the dynamic operation mode. (**b**) Schematic illustration of the measurement system (picomeasure PM3).

Thiolated oligonucleotides, 49 base pairs in length, identified as the Probe in Table 1, were acquired from Integrated DNA Technologies, United States. The obtained probe was immobilized onto the gold-coated surface of the microcantilever using a method described by Alodhayb [15]. Briefly, the cleaned chips of microcantilevers were submerged in the 1 μM thiolated oligonucleotide probe solution for 1 h at room temperature. Probe immobilization was achieved through the formation of a gold–sulfur covalent bond between the thiolated probe and the gold surface of the microcantilever (Figure 1).

### 2.3. DNA Hybridization

In this work, 3 single-stranded DNA sequences, categorized as complementary, partial complementary, and noncomplementary targets (Table 1), were used to study DNA hybridization on the microcantilever platform. These DNA sequences were obtained from the hCoV-19/Canada/LTRI-18/2020 genome. DNA hybridization is the formation of a double-stranded DNA (dsDNA) molecule by base-pairing two single-stranded DNA (ssDNA) sequences. To achieve this, immobilized microcantilever chips were incubated in a 0.3 μM complementary target solution for 30 min. The same protocol was used for the DNA hybridization of partial complementary and noncomplementary targets.

### 2.4. Measurement and Analysis

The resonance frequency of microcantilevers was measured using Picomeasure PM3 (Fourien Inc, Edmonton Canada) in the dynamic measurement module, which included measuring the resonance frequency and the microcantilever phase. The vibration amplitude of the microcantilevers was acoustically excited, and the resonance frequency of the vibrating microcantilevers was measured using a laser (*λ* = 635 nm, power = 1.3 mW) with a spot size of ~30 µm. In the instrument’s optical head, the laser was reflected in a position-sensitive detector that converts the mechanical/optical oscillations into an electrical charge. Figure 1b shows a simple scheme of the Picomeasure instrument’s optical head. For measurement, a sinusoidal signal is swept from 4 kHz to 8 kHz with the amplitude of the excitation signal being 2.5 V. The PM3 software was used to continuously record the frequency measurement, and the data were analyzed separately from the files, which were saved by the instrument in csv format.

Equation (1) represents an established relationship between the resonance frequency and mass of the microcantilever [22].

(1) $$\omega_{0}=\sqrt{\frac{k}{m}}$$

**Figure 2:**
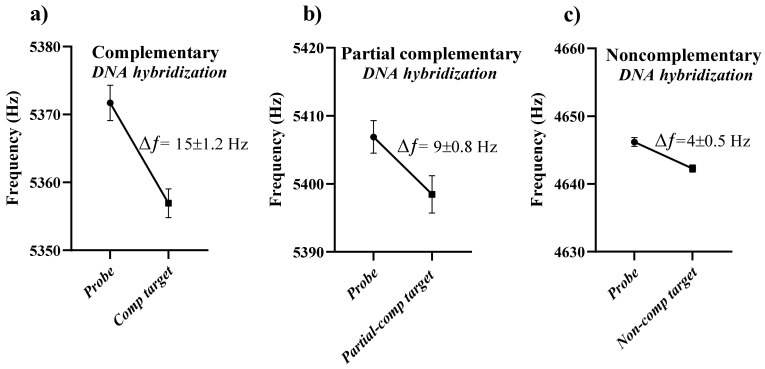
(**a**) The microcantilever’s resonance frequency shift after forming dsDNA by base-pairing of the probe and complementary ssDNA on the microcantilever surface. (**b**) The microcantilever’s resonance frequency shift after partial DNA hybridization of the probe and partial complementary target. (**c**) The resonance frequency shift in response to a mismatch of ssDNA of the probe and noncomplementary target.

**Figure 3:**
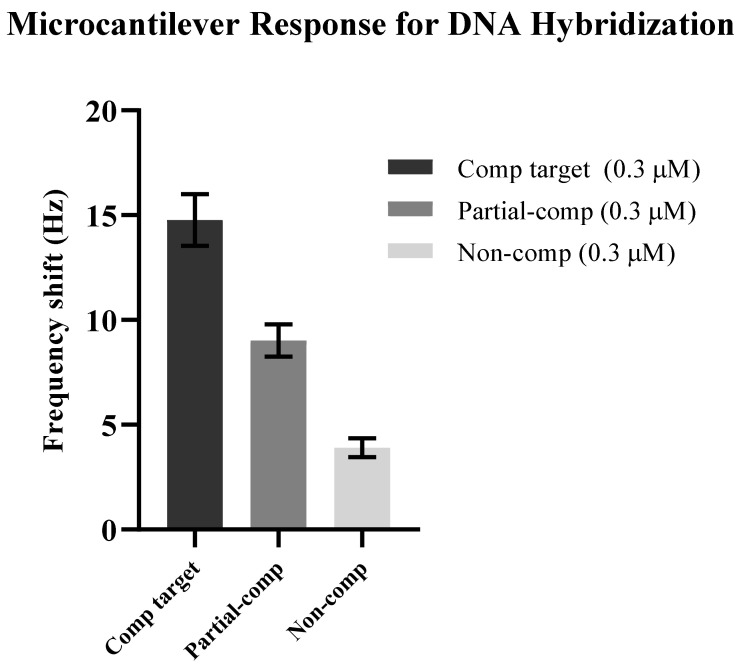
The frequency response of the immobilized microcantilever with 0.3 μM complementary, partial complementary, and noncomplementary targets after hybridization. The resonance frequency shifts of ~15 Hz, 9 Hz, and 4 Hz were observed for complementary, partial complementary, and noncomplementary target DNA, respectively. The presented data are the averages of 3 microcantilever chips used to conduct a single set of experiments.

**Figure 4:**
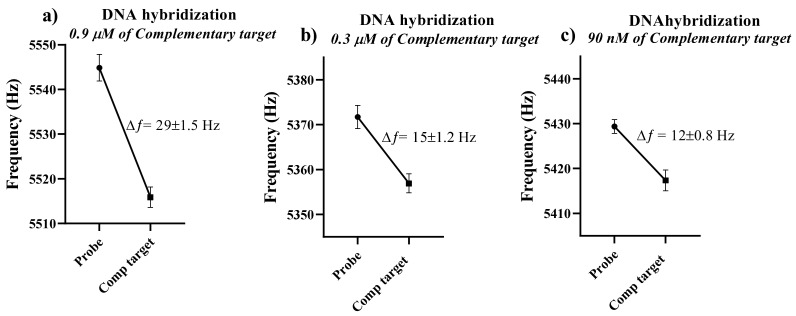
The shift in resonance frequency of the immobilized microcantilever after DNA hybridization with (**a**) 0.9 μM, (**b**) 0.3 μM, and (**c**) 90 nM complementary targets.

**Figure 5:**
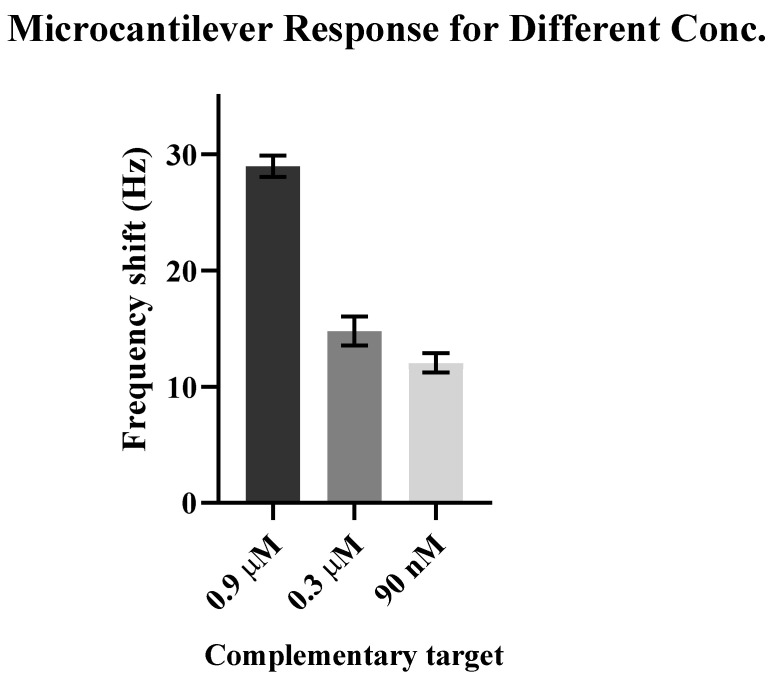
The resonance frequency shift of immobilized microcantilever in response to different concentrations of complementary target DNA. The high concentration induces a large frequency shift.

Here, *k* is the spring constant, $\omega_{0}$ is the resonance angular frequency, and *m* is the effective mass of the microcantilever. Equation (1) shows that the resonance frequency is inversely proportional to the effective mass of the microcantilever. The relationship between the microcantilever’s resonance frequency and effective mass was used to study DNA hybridization on the microcantilever’s surface. In the dynamic mode, the microcantilever’s resonance frequency before and after DNA hybridization was measured, and DNA hybridization was inferred from the shift in resonance frequency because of the mass change. The recorded microcantilever’s resonance frequency was fitted with a Lorentzian curve using the OriginPro software, and the peak parameter of the fitted curve was taken as the microcantilever’s resonance frequency. Furthermore, several complementary sequences with different concentrations (Table 2) were tested to measure the sensitivity of the microcantilever sensors. All measurements for complementary, partial complementary, and noncomplementary DNA hybridizations were taken for 10 min under the same experimental setup. Each experiment was performed using 3 microcantilevers to obtain a statistical comparison of repeatability, and data are presented as mean ± standard deviation (Figure 2, Figure 3, Figure 4 and Figure 5).

## 3. Results and Discussion

The DNA hybridization experiment was conducted to design a microcantilever sensor that can detect the SARS-CoV-2 genomic sequence in a label-free manner. Therefore, a probe was immobilized on the microcantilever’s gold-coated surface, as described in the previous section. Several single-stranded DNA sequences were used in this study to demonstrate the aptitude of microcantilever sensors. Figure 2 shows a change in the microcantilever’s resonance frequency in the dynamic operation mode. Each microcantilever has an individual resonance frequency; therefore, the vertical frequency scale was well-defined to measure the resonance frequency shift of complementary, partial complementary, and noncomplementary DNA hybridization. However, a scale range of 30 Hz was adopted to direct the comparison in the resonance frequency shift. The resonance frequency of the immobilized microcantilever was swept from 5372 Hz to 5357 Hz after DNA hybridization with 0.3 μM of the complementary target (Figure 2a).

Figure 2 shows a resonance frequency shift of ~15 Hz in response to dsDNA formation by base-pairing of the probe ssDNA and complementary target ssDNA. If we assume that the distribution of DNA molecules is uniform on the microcantilever surface and the resonance frequency shift is due to mass variation only, then the estimated complementary target DNA mass is approximately 0.24 µg/ cm^2^ based on a frequency shift of 15 Hz. This corresponds to a target density of 4.3 × 1012 molecules/cm^2^ according to research reports on self-assembled monolayers [23,24,25]. The resonance frequency shift of a microcantilever can be attributed to a number of factors including change in mass, spring constant, stiffness, or damping [26,27,28]; therefore, the calculated DNA mass is not rigorous. The important consideration, however, is that the complementary DNA hybridization caused a resonant frequency shift of ~15 Hz, which is significant to detect COVID-19 using a microcantilever sensor.

In response to DNA hybridization between the probe and 0.3 μM partial complementary target DNA, the resonance frequency of the immobilized microcantilever changed from 5407 Hz to 5398 Hz, showing a shift of 9 Hz (Figure 2b). The substantial reduction of 6 Hz in the resonance frequency shift for the same concentration of the complementary and partial complementary targets explains a small change in the microcantilever mass because of partial DNA hybridization. However, no significant shift in resonance frequency was observed in response to the noncomplementary target. The probe and noncomplementary target were mismatched; therefore, no mass change in the microcantilever was expected because of minimal hybridization. The background variation of ~4 Hz in resonance frequency was noticed for a time of 10 min prior to introducing the DNA sample into the cantilever environment. Therefore, the resonance frequency shift of ~4 Hz was attributed to the background noise signals. Figure 3 shows a direct comparison of the microcantilever’s resonance frequency shift after DNA hybridization of complementary, partial complementary, and noncomplimentary targets.

Figure 4 shows the resonance frequency of the immobilized microcantilever to the complementary target DNA concentration. Three concentrations of complementary target DNA, ranging from 0.9 μM to 90 nM, were used to calculate the microcantilever’s concentration sensitivity. All other parameters, i.e., probe concentration and incubation time, were kept constant while conducting these experiments.

The higher concentration of the complementary target DNA means that more DNA hybridization of the target and probe is expected, which could lead to a large resonance frequency shift. Accordingly, a shift of 29 Hz in the immobilized microcantilever’s resonance frequency was observed in response to 0.9 μM of the complementary target, shifting the resonance frequency from 5545 Hz to 5516 Hz (Figure 4a). This is equivalent to a DNA mass of ~0.46 µg/cm^2^, and a target density of ~8.8 × 10^12^ molecules/cm^2^ based on studies of self-assembled monolayers. The measured microcantilever’s resonant frequency shift in response to 0.3 μM of cDNA was consistent with Figure 3, in which the frequency shift was 15 Hz, resulting from DNA hybridization. In Figure 4c, the corresponding difference in resonance frequency was 12 Hz in the response to 90 nM of the complementary target. The frequency shift of 12 Hz corresponded to 1.8 × 10^12^ target molecules/cm^2^. This shows that the small concentration of complementary target DNA was also effective in changing the microcantilever’s resonance frequency. Figure 5 shows a comparison of the resonance frequency shift to different concentrations of complementary target DNA.

Figure 5 shows that the immobilized microcantilever’s sensor is extremely sensitive, responding to detection of SARS-CoV-2 in the micro-to-nanoscale unit. For cDNA concentrations as low as 90 nM, the functionalized microcantilever sensor was also able to identify genomic sequencing of SARS-CoV-2. Therefore, a nanoscale concentration of SARS-CoV-2 cDNA was required to detect COVID-19 in a label-free manner. Furthermore, our finding demonstrated that a high concentration of complementary target DNA corresponded to a large resonant frequency shift, and it is assumed that an increase in mass from DNA hybridization caused the resonant frequency shift of the microcantilever [23,29]. However, this assumption is not precise because other factors, such as a change in spring constant, stiffness, and damping, might contribute to the resonant frequency shift. The notable observation is that complementary target DNA significantly shifts the resonance frequency compared to noncomplementary target DNA.

## 4. Conclusions

Here, we developed a micromechanical sensor to achieve a quick, efficient, and affordable method for detecting viral RNA in patient samples. The presented method does not require DNA amplification, which is a major bottleneck in RT-PCR testing. However, RNA purification and reverse-transcription of the RNA into cDNA are essential for micromechanical sensing. Our findings demonstrated that micromechanical sensors could successfully discriminate between complementary and noncomplementary ssDNA in a label-free manner. Additionally, complementary ssDNA in the micro-to-nanoscale unit could be detected. Therefore, a nanoscale concentration of cDNA is sufficient to detect COVID-19. The sensing response to partial complementary ssDNA demonstrated that the new variants of SARS-CoV-19 could be successfully detected using microcantilever sensors. In a broader sense, we reason that microcantilever mechanosensing is a promising technique for SARS-CoV-2 detection that could lead to a substantial effort to combat the novel coronavirus pandemic by facilitating rapid detection of SARS-CoV-2 in infected people without expensive and laborious PCR.

## Figures and Tables

**Table 1:** Single-stranded DNA sequences used in this DNA hybridization study.

Oligonucleotide	Sequence and End Modifications	Concentration
Probe	5′-/5ThioMC6-D/CAA CTG GAA CCT CAT CAG GAG ATG CCA CAA CTG CTT ATG CTA ATA TGC T-3′	1 μM (15.4 μg mL^−1^)
Complementary target	5′-/5ThioMC6-D/AGC ATA TTA GCA TAA GCA GTT GTG GCA TCT CCT GAT GAG GTT CCA GTT G-3′	0.3 μM (4.62 μg mL^−1^)
Partial complementary target	5′-/5ThioMC6-D/GTA CTG GCA GAT TAA GCA GTT GTG GCA TCT CCT GAT TAC CGT AAC AGG G-3′	0.3 μM (4.62 μg mL^−1^)
Noncomplementary target	5′-/5ThioMC6-D/GGG TAT CGG TCT ACC TTA TCA AAG ACA TCA AGC TGC AAT GCA CGA TCG-3′	0.3 μM (4.62 μg mL^−1^)

**Table 2:** Complementary sequences used to measure the sensitivity of the microcantilever sensors.

Oligonucleotide	Sequence and End Modifications	Concentration
Probe	5′-/5ThioMC6-D/CAA CTG GAA CCT CAT CAG GAG ATG CCA CAA CTG CTT ATG CTA ATA TGC T-3′	1 μM (15.4 μg mL^−1^)
Complementary target	5′-/5ThioMC6-D/AGC ATA TTA GCA TAA GCA GTT GTG GCA TCT CCT GAT GAG GTT CCA GTT G-3′	0.9 μM (13. 8μg mL^−1^)
Complementary target	5′-/5ThioMC6-D/AGC ATA TTA GCA TAA GCA GTT GTG GCA TCT CCT GAT GAG GTT CCA GTT G-3′	0.3 μM (4.62 μg mL^−1^)
Complementary target	5′-/5ThioMC6-D/AGC ATA TTA GCA TAA GCA GTT GTG GCA TCT CCT GAT GAG GTT CCA GTT G-3′	90 nM (1.38 μg mL^−1^)

## Data Availability

Not applicable.
